# Fertility preservation counseling for women of reproductive age diagnosed with cancer: an integrative review

**DOI:** 10.5935/1518-0557.20230074

**Published:** 2024

**Authors:** Júlia Casemiro Barioni, Thais de Oliveira Gozzo

**Affiliations:** 1 University of São Paulo at Ribeirão Preto College of Nursing, Department Public Health Nursing, Ribeirão Preto - São Paulo, Southeast, Brazil

**Keywords:** directive counseling, fertility preservation, women’s health, antineoplastic agents, nursing, assisted reproductive techniques

## Abstract

This integrative review synthesizes the scientific evidence on fertility preservation counseling prior to oncological treatment for women of reproductive age diagnosed with cancer. Bibliographic research was conducted on databases PubMed, CINAHL, LILACS, EMBASE, Scopus, and Web of Science. The structured search strategy for the review question was “counseling AND antineoplastic agents AND fertility preservation”. The use of controlled descriptors and keywords was adapted for each database. Study selection through the Rayyan platform was independent and blinded. The final sample comprised seven studies emphasizing the importance of clarifying factors related to the risk of infertility due to oncological treatment and fertility preservation techniques, such as success rate, pregnancy rate, cost, available options, and side-effects, as well as discussing the possibilities of adoption and surrogacy. This review provided evidence reinforcing the importance of counseling for fertility preservation, promoting motherhood for women who face oncological treatment. Organized networks linking oncology and reproductive medicine units are crucial to facilitate patient referral between these services and interprofessional communication.

## INTRODUCTION

Cancer is estimated to have been diagnosed in 19.3 million people worldwide in 2020 (International Agency for Research on Cancer [IARC], 2020). In Brazil, 704 thousand new cases of cancer are expected to occur every year from 2023 to 2025 (Santos *et al*., 2023). Women of reproductive age account for 3% to 10% of all cancer diagnoses worldwide (Massarotti *et al*., 2021).

Women of reproductive age diagnosed with cancer require comprehensive fertility and pregnancy care due to treatment being possibly gonadotoxic. After chemotherapy and/or radiotherapy, women present a higher risk of ovarian insufficiency and early menopause, in addition to fibrosis, atrophy, and vascular lesion in reproductive organs (Taylan & Oktay, 2019).

In pelvic radiotherapy, the total dose estimated to increase the risk of early ovarian failure and severe uterine damage is 20 Gy (Tomás *et al*., 2016; Wo & Viswanathan, 2009). In turn, chemotherapy schemes with alkylating agents, such as cyclophosphamide, pose a higher risk of ovarian toxicity and infertility (Zhao *et al*., 2014).

Ovarian changes due to oncological treatment may negatively impact the reproductive plans of women or couples. The continuous discussion of fertility issues throughout survival stages, also before and after cancer therapy, is particularly important for patients who might face changes in their lives, relationship status, or attitude towards building a family (Shen *et al*., 2019).

Oncofertility associates reproductive endocrinology with oncology to increase the access of cancer patients to fertility preservation techniques after oncological diagnosis with the objective of improving the quality of life of cancer survivors (Rashedi *et al*., 2020). Health professionals must inform patients of possible threats to fertility as soon as possible in the process of diagnosis and treatment to provide a broader range of fertility preservation options (ESHRE, 2020; Oktay *et al*., 2018).

Fertility preservation offers women the possibility of having children after oncological treatment; their choice must be considered during counseling by professionals specializing in fertility (ESHRE, 2020; Oktay *et al*., 2018).

Despite the publication of directives emphasizing the importance of fertility preservation counseling as a strategy for fighting the risks of infertility related to oncological treatments, the scientific production on this theme is general, particularly regarding orientation from health professionals.

Identifying the directives for fertility preservation counseling is relevant to improve oncofertility care, given that women of reproductive age diagnosed with cancer do not feel supported in their decision-making (Del Valle *et al*., 2022). Progress in this type of counseling is required for these women to decide their reproductive future based on reliable information. Thus, this study had the objective of analyzing and synthesizing the scientific evidence on fertility preservation counseling prior to oncological treatment for women of reproductive age diagnosed with cancer.

## MATERIAL AND METHODS

### Study design

This study consists of an integrative literature review registered on the Open Science Framework (OSF) platform on 2022, available at https://osf.io/. The study had the following phases: elaboration of the review question, literature search of primary studies, primary data assessment, data analysis, and review presentation (Whittemore & Knafl, 2005).

The guiding question of this study was elaborated using the PICO (Population, Intervention, Control, and Outcome) strategy: P - Women of reproductive age diagnosed with cancer; I - Counseling before a fertility-threatening oncological treatment; C - Not applicable; O - Fertility preservation.

This strategy led to the following guiding question: “Which is the scientific evidence on fertility preservation counseling prior to a fertility-threatening oncological treatment for women of reproductive age diagnosed with cancer?”.

### Search strategy

The search was conducted on September 17, 2023, on the following electronic databases: PubMed, CINAHL, LILACS, EMBASE, Scopus, and Web of Science. The controlled and uncontrolled subject descriptors were identified for structuring a specific search strategy for each database combined with the Boolean operators AND and OR (Table 1).

**Table 1:** Search strategies employed on the databases. Ribeirão Preto, SP, Brazil, 2023.

Databases	Strategy	Results
PubMed	(“counsel”[All Fields] OR “counseled”[All Fields] OR “counselings”[All Fields] OR “counselled”[All Fields] OR “counselling”[All Fields] OR “counseling”[MeSH Terms] OR “counseling”[All Fields] OR “counsellings”[All Fields] OR “counsels”[All Fields] OR (“orient”[All Fields] OR “orientability”[All Fields] OR “orientable”[All Fields] OR “orientate”[All Fields] OR “orientated”[All Fields] OR “orientates”[All Fields] OR “orientating”[All Fields] OR “orientation”[MeSH Terms] OR “orientation”[All Fields] OR “orientations”[All Fields] OR “orientation s”[All Fields] OR “orientation, spatial”[MeSH Terms] OR (“orientation”[All Fields] AND “spatial”[All Fields]) OR “spatial orientation”[All Fields] OR “oriented”[All Fields] OR “orientational”[All Fields] OR “orienting”[All Fields] OR “orients”[All Fields])) AND “Antineoplastic Agents”[All Fields]) OR “Medical Oncology”[All Fields] AND “Fertility Preservation”[All Fields])	455
CINAHL	(Counseling OR Orientation AND “Antineoplastic Agents” OR “Medical Oncology” AND “Fertility Preservation”)	60
LILACS	(Counseling OR Orientation OR Aconselhamento OR Orientação OR Consejería OR Orientación AND “Antineoplastic Agents” OR “Medical Oncology” OR “Agentes Antineoplásicos” OR “Oncologia Médica” OR “Oncología Médica” AND “Fertility Preservation” OR “Preservação da Fertilidade” OR “Preservación De La Fertilidad”)	22
EMBASE	(‘counseling’/exp OR counseling OR ‘orientation’/exp OR orientation) AND (‘antineoplastic agents’/exp OR ‘antineoplastic agents’) OR ‘medical oncology’/exp OR ‘medical oncology’) AND (‘fertility preservation’/exp OR ‘fertility preservation’)	167
Scopus	(Counseling OR orientation AND “Antineoplastic Agents” OR “Medical Oncology” AND “Fertility Preservation” AND (LIMITTO (DOCTYPE, “ar”))	456
Web of Science	(Counseling OR Orientation AND “Antineoplastic Agents” OR “Medical Oncology” AND “Fertility Preservation”)	14

After the searches were conducted, the documents were exported to the Rayyan web app (Ouzzani *et al.,* 2016) to help in the process of organizing and selecting articles, as well as excluding duplicates. The studies were assessed and selected by two independent, blinded reviewers in this app. In the first phase titles and abstracts were screened based on this review’s eligibility criteria. In the second phase the eligible studies were analyzed through full-text reading. Disagreements between the two reviewers were resolved in a consensus meeting. If disagreements persisted, a third reviewer with expertise on the theme was consulted.

### Selection criteria

The inclusion criteria were primary articles dealing the fertility preservation counseling for women of reproductive age published in Portuguese, English, or Spanish, without date restriction. The excluded studies were those involving children, adolescents, or men and those published as editorials, protocols, in annals, as congress abstracts, and reviews.

**Figure 1:**
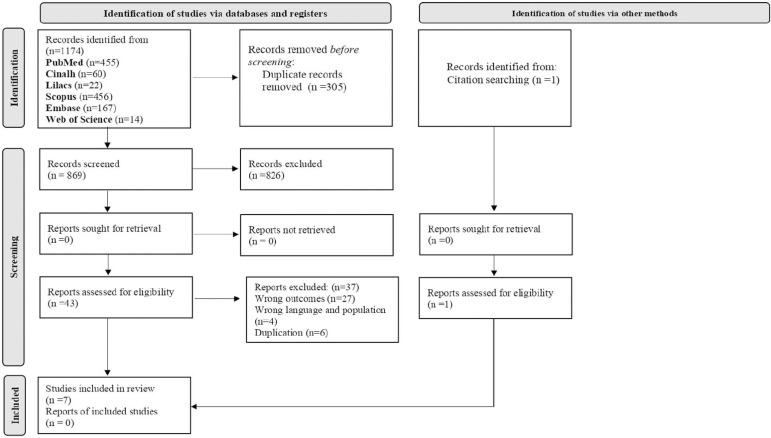
Flowchart of the process of selection of primary studies included in the integrative review based on Preferred Reporting Items for Systematic Review and Meta-Analyses (PRISMA)

Source: Page *et al*. (2021).

The resulting articles amounted to 1,174 and 305 were removed due to duplication. The remaining 869 had their titles and abstracts screened, 43 of which were selected for full-text reading, whereas the other 826 were excluded. After full-text reading, 37 articles were excluded, following the eligibility criteria of this re-view. In addition, one study was identifiedin the reference list and included in the finalsample (handsearching). Thus, seven studies composed this review’s finalsample (Figure 1).

### Data collection instrument

The data from the selected studies were collected in a form containing the following information: authors, year of publication, country, objective, type of study, sample, main results, and conclusions. This phase was conducted by two independent reviewers and disagreements were discussed until a consensus was reached in the meetings.

### Data analysis

The type of study was identified according to the classification provided by the authors of the studies included in this review. This information was not found in two of the studies, which were classified as qualitative.

The methodological quality of the included articles was assessed through instruments in accordance with the type of study proposed by the Joanna Briggs Institute’s (JBI) scientific committee. The JBI instruments are composed of questions to be answered by the reviewer with “yes”, “no”, or “unsure”. The questions assess the internal validity and risk of bias of the studies. They do not propose a score system for study assessment, but a higher number of questions replied with “yes” indicates a better methodological quality (Moola *et al.,* 2020).

The instruments were independently applied by two reviewers and disagreements were solved in a consensus meeting between the reviewers. The data were analyzed and synthesized descriptively.

## RESULTS

The flowchart for the selection process of primary stud-ies included in the integrative review (IR) is presented in Figure 1. Thus, out of the 1174 publications identified in the databases (registers), after the application of the eligibil-ity criteria, 43 primary studies were selected for full-text reading, 37 articles were excluded, one study was identifiedin the reference list and included in the final sample and 7 comprised the review sample.

Table 2 presents the descriptive synthesis of the primary studies included in this review by author, year of publication, country, language, type of study, sample, objective, and main results.

**Table 2:** Descriptive synthesis of the included studies comprising the final sample of this integrative review (n=7).

Author, year, country, and language	Type of study/sample	Objective	Main results
Peate *et al*., 2011 Australia English	Prospective study (authors) n=111	To explore knowledge related to fertility, decisional conflict, preference of information on fertility, and decision-making of female breast cancer patients.	Out of 111 participants, 74% wished to receive information about the effects of oncological treatment on fertility. The lower the knowledge, the higher the decisional conflict on fertility preservation (odds ratio [OR] 0.57; 95% CI, 0.44 to 0.73; *p*<.001).
Garvelink *et al*., 2015 Netherlands English	Qualitative study (authors) n=34	To explore the experiences of providing information on gonadotoxic effects, fertility preservation, and the decision-making process of female cancer survivors.	The participants report the need for further clarification of fertility preservation procedures and the side-effects of the employed techniques, in addition to alternative options for having children (such as adoption) after chemotherapy.
Srikanthan *et a*l., 2019 Canada English	Qualitative study (authors) n=50	To explore the experiences of breast cancer survivors with fertility during the diagnosis	Out of fifty participants, 62% would not submit to fertility preservation treatment unless the available technology offered more than 50% of success probability. For 72%, financial issues limited the search for fertility preservation, even when desired. Clarification of the effects of the treatment on fertility after its conclusion and of other strategies, such as surrogacy and adoption, is required.
Speller *et al*., 2019a Canada English	Qualitative study n=28	To create and test the “Begin Exploring Fertility Options, Risks, and Expectations” (BEFORE) decision aid for premenopausal breast cancer patients	The BEFORE decision aid is an online tool to be used by patients and professionals and applicable to clinical practice (Alpha testing) as a complement to counseling clinical consultations about fertility preservation and decision-making.
Speller *et al*., 2019b Canada English	Qualitative study (authors) n=16	To assess six resources/materials aimed at oncofertility decision support for breast cancer patients and health providers	The following themes were pointed out by patients and professionals as necessary information to be included in the resources/materials: age and treatment-related fertility decline, post-treatment pregnancy rates made possible by fertility preservation techniques, and the health of children born of surviving patients through fertility preservation techniques.
Ehrbar *et al*., 2019 Sweden and Germany English	Randomized clinical trial (authors) n=79	To understand whether the use of an online fertility preservation decision aid in addition to counseling can reduce decisional conflict in fertility preservation	The women who received counseling and used the decision aid presented lower decisional conflict compared to the group which received counseling alone (*p*=0.008; M=12.15, SD=4.38; 95% CI, 3.35-20.95 and *p*=0.043; M=9.35, SD=4.48; 95% CI, 0.31-18.38).
van den Berg *et al.*, 2021 Netherlands English	Qualitative study n=53	To develop and test decision aids for fertility preservation of female cancer patients of reproductive age	Survivors and professionals consider decision aids as very useful in the process of decision-making and rated them as 8.5 (1-10 scale). The decision aid must be developed by cancer type and contemplate adoption, surrogacy, and the steps to getting pregnant after cancer treatment.

In the assessment of qualitative studies (n=5) with the JBI Critical Appraisal Checklist for Qualitative Research instrument, out of the 10 questions composing the checklist, four studies had 7 responses with “yes” and one study presented “yes” 8 times, as can be seen in Table 3.

**Table 3:** Methodological assessment of the primary studies through the JBI Critical Appraisal Checklist for Qualitative Research tool.

Qualitative study	Q1	Q2	Q3	Q4	Q5	Q6	Q7	Q8	Q9	Q10	Total (Yes)
Speller *et al*. (2019a)	U	Y	Y	Y	Y	N	N	Y	Y	Y	7
Garvelink *et al*. (2015)	N	Y	Y	Y	Y	N	N	Y	Y	Y	7
Srikanthan *et al*. (2019)	N	Y	Y	Y	Y	N	N	Y	Y	Y	7
Van den Berg *et al*. (2021)	Y	Y	Y	Y	Y	N	N	Y	Y	Y	8
Speller *et al*. (2019b)	U	U	U	Y	Y	Y	Y	Y	Y	Y	7

Q1 = Is there congruity between the stated philosophical perspective and the research methodology?, Q2 = Is there congruity between the research methodology and the research question or objectives?, Q3 = Is there congruity between the research methodology and the methods used to collect data?, Q4 = Is there congruity between the research methodology and the representation and analysis of data?, Q5 = Is there congruity between the research methodology and the interpretation of results?, Q6 = Is there a statement locating the researcher culturally or theoretically?, Q7 = Is the influence of the researcher on the research, and vice-versa, addressed?, Q8 = Are participants, and their voices, adequately represented?, Q9 = Is the research ethical according to current criteria or, for recent studies, and is there evidence of ethical approval by an appropriate body?, Q10 = Do the conclusions drawn in the research report flow from the analysis, or interpretation, of the data?, Y = Yes, N = No, U = Unclear.

In the assessment of the randomized study (n=1) with the JBI Critical Appraisal Tool for Assessment of Risk of Bias for Randomised Controlled Trials, out of the 13 questions composing the checklist, 10 had “yes” as a response, as can be observed in Table 4.

**Table 4:** Methodological assessment of the primary studies through the JBI Critical Appraisal Tool for Assessment of Risk of Bias for Randomised Controlled Trials.

Randomized study	Q1	Q2	Q3	Q4	Q5	Q6	Q7	Q8	Q9	Q10	Q11	Q12	Q13	Total (Yes)
Ehrbar *et al*. (2019)	Y	Y	Y	Y	U	U	Y	Y	Y	Y	U	Y	Y	10

Q1 = Was true randomization used for assignment of participants to treatment groups?, Q2 = Was allocation to treatment groups concealed?, Q3 = Were treatment groups similar at the baseline?, Q4 = Were participants blind to treatment assignment?, Q5 = Were those delivering the treatment blind to treatment assignment?, Q6 = Were treatment groups treated identically other than the intervention of interest?, Q7 = Were outcome assessors blind to treatment assignment?, Q8 = Were outcomes measured in the same way for treatment groups?, Q9 = Were outcomes measured in a reliable way?, Q10 = Was follow up complete and if not, were differences between groups in terms of their follow up adequately described and analysed?, Q11 = Were participants analysed in the groups to which they were randomized?, Q12 = Was appropriate statistical analysis used?, Q13 = Was the trial design appropriate and any deviations from the standard RCT design (individual randomization, parallel groups) accounted for in the conduct and analysis of the trial?, Y = Yes, N = No, U = Unclear.

In the assessment of the prospective study (n=1) through the JBI Critical Appraisal Tool for Assessment of Risk of Bias for Cohort Studies, out of 11 questions comprising the checklist, the study had “yes” as a response 5 times, as can be observed in Table 5.

**Table 5:** Methodological assessment of the primary studies through the JBI Critical Appraisal Tool for Assessment of Risk of Bias for Cohort Studies.

Prospective study	Q1	Q2	Q3	Q4	Q5	Q6	Q7	Q8	Q9	Q10	Q11	Total (Yes)
Peate *et al*. (2011)	U	U	U	U	U	U	Y	Y	Y	Y	Y	7

Q1 = Were the two groups similar and recruited from the same population?, Q2 = Were the exposures measured similarly to assign people to both exposed and unexposed groups?, Q3 = Was the exposure measured in a valid and reliable way?, Q4 = Were confounding factors identified?, Q5 = Were strategies to deal with confounding factors stated?, Q6 = Were the groups/participants free of the outcome at the start of the study (or at the moment of exposure)?, Q7 = Were the outcomes measured in a valid and reliable way?, Q8 = Was the follow up time reported and sufficient to be long enough for outcomes to occur?, Q9 = Was follow up complete, and if not, were the reasons to loss to follow up described and explored?, Q10 = Were strategies to address incomplete follow up utilized?, Q11 = Was appropriate statistical analysis used?, Y = Yes, N = No, U = Unclear.

## DISCUSSION

This study synthesized the scientific evidence related to fertility preservation counseling for women of reproductive age diagnosed with cancer, verifying that these patients wish to receive information on the fertility preservation procedures, such as cost, risks, adverse events, and success and pregnancy rates. The studies show that the possibility of becoming a mother is important for many women after a cancer diagnosis, not necessarily by giving birth, but resorting also to other strategies, such as adoption and surrogacy.

In a whole care model for women of reproductive age diagnosed with cancer, orientation on gonadotoxic effects must be provided as early as possible, before oncological therapy is started. This can be provided by the clinical care team, which must then refer the patient to a professional specializing in fertility for fertility preservation counseling (ESHRE, 2020; Kufel-Grabowska *et al*., 2022).

The women and the health professionals manifested different concerns related to fertility preservation. The professionals reflected on the possible information overload during diagnosis upon premature referral to fertility specialists (Speller *et al*., 2019a). In addition to lacking knowledge on fertility preservation, they also present questions on who must be the responsible professional for discussing this procedure, considering that this is a difficult theme and the professionals believe that it makes female patients worried (Van den Berg *et al*., 2021). The women wish to discuss this theme because it generates positive feelings, such as the hope of becoming mothers, and have demonstrated discomfort with having little time to make this decision before oncological therapy (Daly *et al*., 2019; Garvelink *et al*., 2015).

Such aspects compromise counseling quality and subsequent decision-making in fertility preservation. Therefore, training programs are fundamental for health professionals to enhance their knowledge of this theme and apply it in their daily work (Takeuchi *et al*., 2018). Training on oncofertility competences helps establishing roles and improving the implementation of guidelines and access to care (Anazodo *et al*., 2019).

In the face of barriers to professionals regarding oncofertility care, strategies were compiled to improve this aspect of care in clinical practice, such as the development of educational tools directed at patients (leaflets, decision aids), professional education, and the inclusion of oncological nurses with a defined role in fertility preservation (Van den Berg *et al*., 2019).

Decision aids are tools which provide information and clarify the agreement between decisions and personal values; they may complement counseling, as observed in three studies included in this review (Speller *et al*., 2019a, 2019b; Van den Berg *et al*., 2021). These resources, used in different contexts of the health area, help in the decision process by providing information on available options, risks, and benefits associated to each of the fertility preservation techniques, minimizing uncertainties (Gonçalves, 2021; Stacey *et al*., 2017). These may be printed and/or online versions, available in different countries and languages. As of this review’s literature search, no Brazilian version was found.

Decisional conflict is defined as uncertainty on whether to submit to the fertility preservation procedure. In a study assessing 111 female cancer patients of reproductive age, 63.1% presented high decisional conflict (Peate *et al.,* 2011). Similar data was found in a study with 155 women, 62.7% of whom also presented high decisional conflict (Müller *et al*., 2018).

The choice for preserving fertility must be based on biopsychosocial principles, so that the health professional may help in the process of decision-making through integrated and interdisciplinary actions. Oncofertility nurse navigators may coordinate the fertility preservation counseling team, articulating care between cancer treatment centers and fertility services, in addition to the skills to identify women who will be submitted to treatment which might affect fertility and who are eligible for its preservation (Keim-Malpass *et al*., 2018; Semler & Thom, 2019; Zwingerman *et al*., 2020).

In the analyzed studies, the technique of cryopreservation was the most frequent and preservation of ovarian, egg, or embryonic tissue varied from 24 to 76.9% (Garvelink *et al.*, 2015; Srikanthan *et al*., 2019; Van den Berg *et al*., 2021). The literature shows that cryopreservation of eggs and embryos is a consolidated technique with a favorable gestational outcome among reproductive-age women with an indication for oncological treatments considered to be gonadotoxic (ESHRE, 2020).

Counseling should discuss the possibility of surrogacy as an alternative option for motherhood, particularly for women who are temporarily or permanently unable to carry a pregnancy after oncological treatment or choose not to do so, particularly in cases of pelvic radiotherapy (ESHRE, 2020). Adoption is also an alternative for building a family after cancer treatment depending on the cultural and legal characteristics of each country (Rashedi *et al*., 2020).

The high financial cost of the procedures creates difficulties in choosing fertility preservation (Anazodo *et al*., 2019; Silva *et al*., 2021; Srikanthan *et al*., 2019). The costs vary with the laws regulating the health systems of each country, regardless of their status as developed or in development. In Canada, cryopreservation of eggs and embryos varies from being free of charge to costing thousands of Canadian dollars; despite Canada’s public and universal healthcare system, coverage of procedures differs among provinces (Brandão, 2019; Speller *et al*., 2019a). In Brazil, the same procedure costs twenty minimum wages and is neither provided by the Unified Health System (*Sistema Único de Saúde* - SUS) nor listed as a procedure by the National Supplementary Health Agency (*Agência Nacional de Saúde Suplementar* - ANS).

The limitations of this study are related to a lack of data regarding the role of relatives and partners in the process of fertility preservation decision-making, given that these are essential figures in the perspective of whole care.

This is added to a lack of clarity on the singularities of the professional classes in the process of fertility preservation counseling and the fact that most studies comprising this review have been conducted with survivors.

Recently diagnosed female cancer patients are emphasized to present different needs for information; however, the survivors presented an encompassing view of treatment stages and registered fundamental aspects to be discussed during counseling. This situation might not have been possible only with studies discussing recently diagnosed female patients, given that initially the worries about the severity of this diagnosis leads to a higher priority of survival, and fertility issues are not considered during diagnosis.

Other limitations include the fact that gray literature was not considered, in addition to language restrictions. Data analysis and synthesis was descriptive, which may have led to a bias in the elaboration of the results. On the other hand, the search for primary studies was conducted on relevant databases for the health and nursing fields. The rigor in the conduction of this synthesis was strengthened by the methodological quality assessment of the studies with tools elaborated by the Joanna Briggs Institute.

## CONCLUSION

The results of this review emphasize the importance of creating or improving educational resources and decision aids to enhance the process of fertility preservation counseling, minimizing decisional conflict. Women wish to understand different fertility preservation techniques and other possibilities for becoming mothers, such as adoption and surrogacy.

This review generated evidence that reinforces the importance of fertility preservation counseling, providing women facing oncological treatment with the possibility of raising children. Organized networks between oncology and reproductive medicine units are crucial to facilitate the referral of patients among services and interprofessional communication.

The study pointed out a lack of data regarding the role of relatives and partners in the process of fertility preservation decision-making, given that these are essential figures in the perspective of whole care. It is suggested that further research be carried out in order to better understand this gap.
